# Preliminary Rasch analysis of the multidimensional assessment of interoceptive awareness in adults with stroke

**DOI:** 10.1371/journal.pone.0286657

**Published:** 2023-06-02

**Authors:** Jena Blackwood, Sydney Carpentier, Wei Deng, Ann Van de Winckel

**Affiliations:** 1 Division of Rehabilitation Science, Department of Rehabilitation Medicine, Medical School, University of Minnesota, Minneapolis, Minnesota, United States of America; 2 Division of Physical Therapy, Division of Rehabilitation Science, Department of Rehabilitation Medicine, Medical School, University of Minnesota, Minneapolis, Minnesota, United States of America; Kio University, JAPAN

## Abstract

**Purpose:**

The Multidimensional Assessment of Interoceptive Awareness (MAIA) measures interoceptive body awareness, which includes aspects such as attention regulation, self-regulation, and body listening. Our purpose was to perform a preliminary validation of the MAIA in adults with stroke using Rasch Measurement Theory.

**Methods:**

The original MAIA has 32 items that measure interoceptive sensibility, which is an aspect of body awareness. We performed a preliminary analysis with Rasch Measurement Theory to evaluate the unidimensionality and structural validity of the scale. We investigated overall fit to assess unidimensionality, person and item fit, person separation reliability, targeting, local item dependence, and principal components analysis of residuals.

**Results:**

Forty-one adults with chronic stroke (average 3.8 years post-stroke, 13 women, average age 57±13 years) participated in the study. Overall fit (χ ^2^ = 62.26, p = 0.26) and item fit were obtained after deleting 3 items and rescoring 26 items. One participant did not fit the model (2.44%). There were no floor (0.00%) or ceiling effects (0.00%). Local item dependence was found in 42 pairs. The person separation reliability was 0.91, and the person mean location was 0.06±1.12 logits.

**Conclusions:**

The preliminary structural validity of the MAIA demonstrated good targeting and reliability, as well as unidimensionality, and good item and person fit in adults with chronic stroke. A study with a larger sample size is needed to validate our findings.

## Introduction

Worldwide, approximately 15 million people suffer a stroke each year, and roughly, half are left with permanent disabilities impeding upper limb use in daily activities, balance, and ambulation as well as influencing mental health and coping strategies [1–9]. Additionally, adults with stroke can have body awareness deficits and, more generally, body representation deficits [10–12]. Body awareness refers to a perceptual understanding and awareness of (i) proprioception: body position and movement sense and how those body parts are situated in peripersonal space, (ii) exteroception: visual, tactile, auditory signals locations within the peripersonal space; and (iii) interoception: awareness of internal body states [13–18]. Body awareness (also called body schema) is one type of mental body representations [11, 19, 20]. Body representations inform and accurately update information about body positions and movements in space at any moment in time, in order to guide motor actions correctly [21–23].

Recent literature has shown that 81% of adults with vascular brain injuries exhibit deficits in at least one type of mental body representations [12]. Data from lesion studies on brain-damaged patients with body representation disorders also support the pivotal role of interoceptive processing in body representations [23–28]. Our prior work demonstrated that exercises from a physical therapy approach that helps restore body representations—called Cognitive Multisensory Rehabilitation -, activates the parietal operculum and the insula [29–31] and that Cognitive Multisensory Rehabilitation restores parietal operculum connectivity with other brain areas concurrent with sensorimotor recovery post stroke lasting at least 1 year [11]. As part of the ongoing body of research, it is essential to use outcome measures that support an accurate assessment of body awareness in adults with stroke, especially when body awareness is assessed in intervention studies aimed at improving body awareness in this population.

Interoception, as part of body awareness, is typically assessed in two ways: interoceptive accuracy (e.g., heartbeat detection accuracy), and interoceptive sensibility, which is self-reported sensory awareness in response to “physiological states, processes (including pain and emotion), and actions (including movement)” [32, 33]. Interoceptive sensibility is typically tested with the Multidimensional Assessment of Interoceptive Awareness (MAIA), which includes aspects such as attention regulation, self-regulation, and body listening [16, 32–38]. Aspects of the MAIA related to monitoring body state were associated with differential neural activation, notably in the insula, when healthy adults were attending to body sensations [38]. The MAIA is a 32-item self-report instrument [37]. More details on the conceptual construct of this scale can be found in Mehling et al. (2012) [37]. Items are scored on a Likert scale between “0 = Never” to “5 = Always”, with unlabeled interim integers [37]. A higher score indicates “more positive interoceptive body awareness” [37]. The MAIA was developed with the intention to differentiate between experienced and inexperienced mind-body practitioners, to measure changes in participants’ outcomes after mind-body therapies, and to “differentiate between anxiety-driven hypervigilance and mindful attention styles” [36, 37, 39]. As such, the MAIA has been heavily utilized for body awareness training measurement in varied populations, but to the best of our knowledge, it has been used in only one previous observational study with adults with stroke [9, 40]. At the time of publication, it has been translated into over 24 languages [41].

Reliability, internal consistency, correlations with related construct measures and between subscales, and confirmatory factor analysis were performed on the MAIA during its initial publication [37]. Mehling et al. (2012) originally demonstrated that most MAIA subscales had moderate to strong correlations with each other, but the subscales “Not Distracting” and “Not Worrying” subscales showed only small to moderate correlations to the other subscales [37]. The internal consistency, as measured by Cronbach’s alpha, was below 0.70 for the subscales “Noticing”, “Not-Distracting”, and “Not-Worrying” and was confirmed in a subsequent study [40]. The other subscales ranged from 0.79 to 0.87 [37, 42–55]. The confirmatory factor analysis confirmed the multidimensionality of the scale [37]. While the MAIA has been evaluated for reliability and validity in healthy adults [37, 44], to our knowledge, the MAIA has not yet been investigated for structural validity in adults with stroke. It is important to know whether the items are measuring what they are supposed to measure in a specific clinical population prior to using the scale as an outcome measure for intervention studies, and prior to that, whether it would be worthwhile to doing a larger validation study in adults with stroke. Therefore, our aim is to perform a preliminary study to evaluate the structural validity of this scale with Rasch analysis in adults with stroke.

Rasch Measurement (RM) Theory can be used to assess the unidimensionality and the structural validity of a scale. The model of RM Theory is a probabilistic model stating persons with a higher ability on a certain trait (in this case, interoceptive body awareness) would have a higher probability of obtaining a higher score on the items. RM Theory can therefore be used to verify whether the items fit the probabilistic mathematical Rasch model. With this analysis, the scale is transformed from an ordinal to an interval measurement which improves instrument precision [56–62]. Structural validity and unidimensionality are evaluated through the overall fit of the scale, the item and person fit, threshold order of scoring categories within each item, person separation reliability (PSR), targeting, floor and ceiling effects, mean error variance, principal components analysis of residuals (PCAR), and local item dependence (LID) with residual correlations [58, 59, 63, 64]. The aim of this study was to perform a preliminary Rasch analysis of the MAIA to evaluate whether doing a full-scale structural validity in community-dwelling adults with chronic stroke in the future would be worthwhile. To the best of our knowledge, the MAIA has not been validated with RM Theory before.

## Methods

### Participants

For this cross-sectional design, potential participants contacted the Brain Body Mind Lab members after seeing the study flier on the University Campus and in clinics, or through postings on the University websites. We included community-dwelling adults between 18 and 99 years of age who had an ischemic or hemorrhagic stroke and who were medically stable, English-speaking, and able to consent. We excluded participants with cognitive impairments (Mini-mental State Exam-brief version, <13/16) [65], severe aphasia [66] apraxia [67–69], or other medical conditions that would preclude participation in the study. The study was approved by the University of Minnesota’s Internal Review Board (IRB#STUDY00000821). The study was performed in accordance with the Declaration of Helsinki. All participants signed informed consent on paper and filled out the MAIA on paper.

### Data collection

We collected information on age, the hemispheric side of the stroke, time after stroke, and whether the stroke was ischemic or hemorrhagic. We assessed pain with the Numeric Pain Rating Scale (NPRS), depression with the Patient Health Questionnaire (PHQ-9), and stroke-related neurologic deficits with the NIH stroke scale (NIHSS) [65, 70, 71]. Participants also indicated if they have done any breathing exercises in the past (as a lifestyle choice); if they were currently performing breathing exercises regularly at home, and if they had past body awareness training experiences such as dance, martial arts, Tai Chi, Qigong, yoga, Pilates, or other body awareness training.

The MAIA was our main outcome, with 32-item self-report items related to interoceptive body awareness [37]. The scoring range was between “0 = Never” to “5 = Always”, with unlabeled interim integers. We decided to investigate the unidimensionality of the MAIA by analyzing all items together rather than performing a Rasch analysis per subscale. If items had reverse scoring, they were recoded before the Rasch analysis so that higher scores would imply higher interoceptive awareness across all items.

### Statistical analysis

Preliminary Rasch analysis was performed using the Rasch Unidimensional Measurement Model (RUMM) 2030 software (RUMM Laboratory, Perth, WA) using a partial credit model and rating scale polytomous model to analyze structural validity and unidimensionality. We report on the following outputs, provided by RUMM2030: The overall fit of the scale, the item and person fit, threshold order of scoring categories within each item, PSR, targeting, floor and ceiling effects, mean error variance, PCAR, and LID with residual correlations [58, 59, 63, 64]. Differential item functioning (DIF) can be calculated when subgroups have a sample size of at least 200, and when the subgroups are of approximately equal size [72]. Given that this is a preliminary study with a smaller sample size, DIF was not calculated.

In terms of the above-mentioned outputs from the RUMM2030, a “threshold” is the point at which adjacent categories have the same likelihood of being selected [73, 74]. Disordered thresholds reveal that the logical estimated order of the scale construct is not measured appropriately by the item response categories and that respondents may have had difficulty differentiating between categories and/or that not all categories were used [59, 74–78].

Fit statistics indicate the appropriateness of person and item fit to the Rasch model [74]. Person and item fit residuals greater than +2.50 indicate misfitting items or persons, and less than -2.50 indicate redundant items (i.e., overfit). Significant p-values for item fit are calculated with Bonferroni correction [59].

PSR differentiates the person’s ability levels of the trait for research or clinical purposes, with PSR > 0.90 allowing the researcher or clinician to make decisions for individuals and ≥ 0.70 to make group decisions [59, 79, 80]. Floor and ceiling effects above 15% are considered problematic [60, 73]. The average person location within a range of -0.50 and 0.50 logits of average item location (by default positioned at 0 logits) indicates adequate assessment targeting [59, 81]. PCAR assesses the random variance in residuals, and if the variance is due to the underlying trait rather than other components [59, 82]. LID is found when an item pair shares a greater degree of content compared to other assessment items [63, 64]. Standard residual correlations of 0.20 or greater than the average of the standard residual item correlation indicate the presence of LID [59, 83].

## Results

Forty-one adults (average age 57±13.65 years, average time post-stroke 3.79±2.85 years) with unilateral ischemic brain lesions resulting in upper limb impairment participated in the study between September 27, 2017, and February 28, 2020. All participants were residing in the community and had chronic stroke. At the time of testing, none of them were involved in a rehabilitation program. Participant demographics and stroke characteristics are detailed in Table 1. Of note, neglect and sensory deficit are frequently present in adults with stroke, and may affect MAIA testing, specifically the questions related to attention regulation and body listening. Most of our participants did not have neglect (average 0.44±0.59 with score 0 being normal function) or sensory deficits (0.37±0.58 with 0 being normal function) on the sub-scores of the National Institutes of Health Stroke Scale (NIHSS). Furthermore, insula activation is known to be associated with attending to body sensations [38]. In our sample, 56% of our adults had lesions in the middle cerebral artery region of the brain but we were unable to discern whether or not insula functions or connections to and from the insula were affected by the brain lesions.

**Table 1:** Demographic and clinical characteristics of study participants.

	Adults with stroke (n = 41)
Age (mean ± standard deviation; range)	57±13 (25–81)
Sex	
Men	28
Women	13
Type of Stroke	
Ischemic	31
Hemorrhagic	9
Unknown	1
Hemisphere of Stroke	
Left	29
Right	12
Time since stroke in years (mean ± SD; range)	3.79 ± 2.85 (.50–10.50)
Location of brain injury (n)	
• Basal ganglia	9
• MCA infarct	23
• other locations (ACA infarct, pons, brainstem, …)	6
• unknown	3
Current participation in breathing exercises (yes; no)	17; 24
Past participation in breathing exercises (yes; no)	18; 23
Past participation in body awareness training (yes; no)	19; 22
Current pain (NPRS) (yes; no)	21; 20
Depression (PHQ-9 >10) (yes; no)	6; 35
MMSE (mean ± SD)	15.24 ± 0.83
MAIA (mean ± SD)	25.17 ± 5.23
Revised Body Awareness Scale (mean ± SD)	14.56 ± 6.02
Warwick-Edinburgh Mental Well-being Scale (mean ± SD)	54.41 ± 8.39
PBE-QAG (mean ± SD)	10.39 ± 6.33
NIHSS (mean ± SD) NIHSS sensory function (mean ± SD)	2.78 ± 2.440.44 ± 0.59
NIHSS neglect (mean ± SD)	0.37 ± 0.58
MESUPES, upper limb motor function (mean ± SD)	31.66 ± 21.54
Exteroception index, thumb, palm (total 6)	5.07 ± 1.46
Proprioception wrist, index (total 8)	6.85 ± 2.30
Stereognosis (total 6 objects)	3.73 ± 2.52
Apraxia (TULIA test) (mean ± SD)	11.34 ± 1.35

MESUPES = Motor Evaluation Scale for Upper Extremity in Stroke Patients

MMSE = Mini-Mental State Examination, NIHSS = National Institutes of Health Stroke Scale, NPRS = Numeric Pain Rating Scale, PBE-QAG = The Physical Body Experiences Questionnaire Simplified for Active Aging, PHQ-9 = Patient Health Questionnaire–9, NIHSS = National Institutes of Health Stroke Scale Score, MMSE = Mini-Mental State Examination

The iteration table (S1 Table) details the step-by-step process of RM Theory analysis for the MAIA. More detailed explanations are below.

Upon initial analysis, 29 of the 32 items on the MAIA presented with disordered thresholds. Over the course of 5 iterations of rescoring, all 29 items were rescored by collapsing response categories. Nine of the original items were collapsed from the original scoring format [0 1 2 3 4 5] to dichotomous response categories [0 0 0 0 1 1], with the other rescored items varying on the spectrum of 3 to 4 collapsed response categories ([0 0 0 0 1 2], [0 0 0 1 1 2], [0 0 0 1 2 3], [0 0 1 1 2 2], [0 0 1 2 2 3]). Only items 14 and 15 retained the original scoring format [0 1 2 3 4 5].

Following rescoring, item 5 “I do not notice (I ignore) physical tension or discomfort until they become more severe.” displayed misfit (Fit Residual = 4.19; p = 0.0001) and was subsequently removed. Next, item 16 “I can maintain awareness of my whole body even when a part of me is in pain or discomfort.” and item 23 “When I feel overwhelmed, I can find a calm place inside.” were removed because they demonstrated a Guttman-like response pattern covering a wide logit-range from -9 logits to 1 logit as displayed (S1 and S2 Figs). Next, items 11, 12, 18, 20, 26, 30, and 31 all displayed small thresholds in the middle scoring categories of each item’s scale and were rescored to improve fit to the model as indicated in the iteration table (S1 Table). Finally, item 25 was rescored due to a small threshold in the middle scoring categories. The resulting item location in logits for all items, after the above steps were completed, is listed in the S2 Table. There were no remaining misfitting or overfitting items (S2 Table).

**Fig 1:**
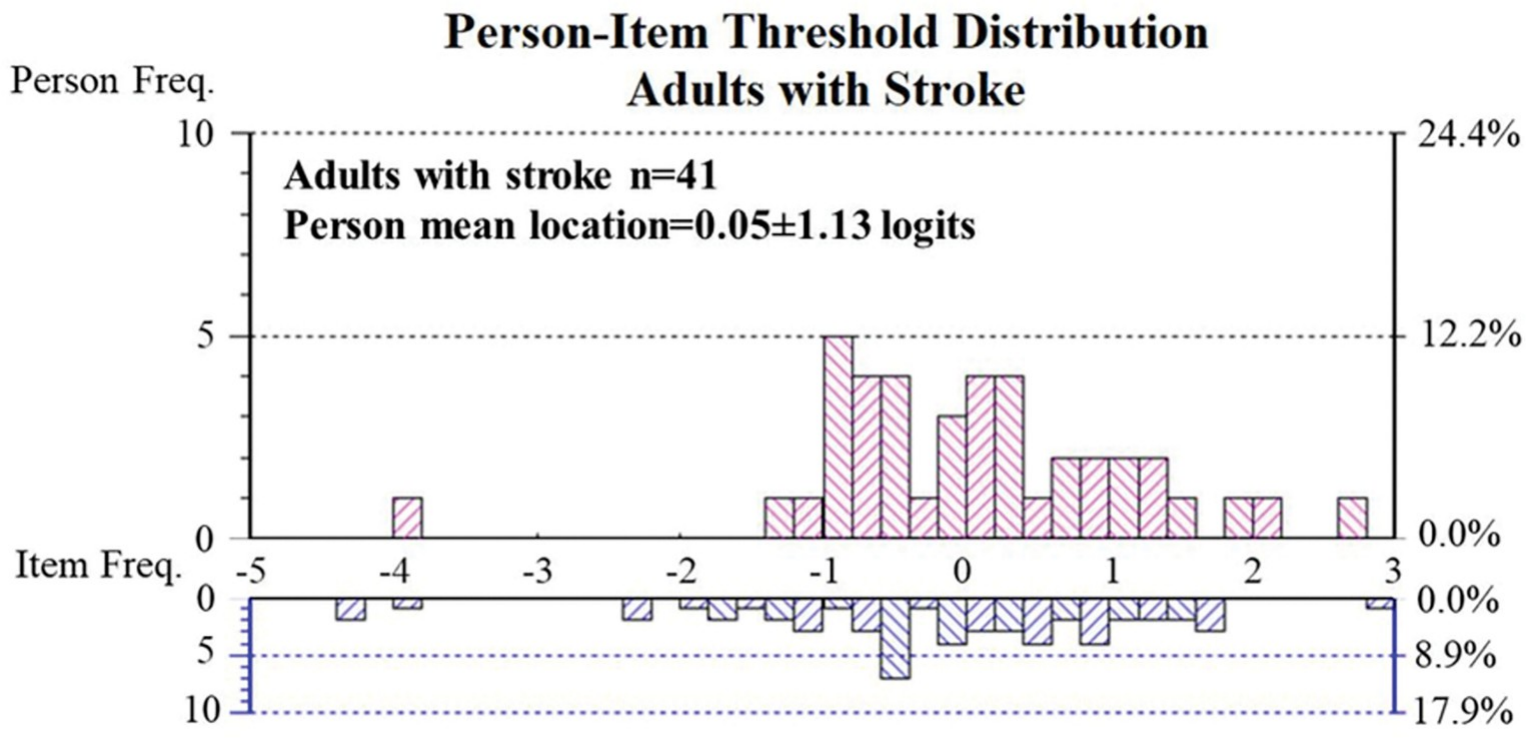
Person-item threshold distribution in adults with stroke. The X-axis logit ruler represents item difficulty and respondent ability. The blue histograms show item difficulty level frequencies, whereas the pink histograms represent the frequencies of the respondent’s ability level of interoceptive body awareness. Higher levels of body awareness are indicated by higher logit values.

The person mean location was 0.05±1.13 logits indicating that the MAIA item difficulty was well-targeted for this population (Fig 1). There was no floor (0.00%) or ceiling effect (0.00%). We found that only 1 of the 41 participants displayed misfit (2.44% of the total group). The PSR was 0.91 indicating that the scale, if this is confirmed in a bigger sample size, can be used for individual decision-making. The mean error variance was 0.12 logits, which is a small error estimate.

**Fig 2:**
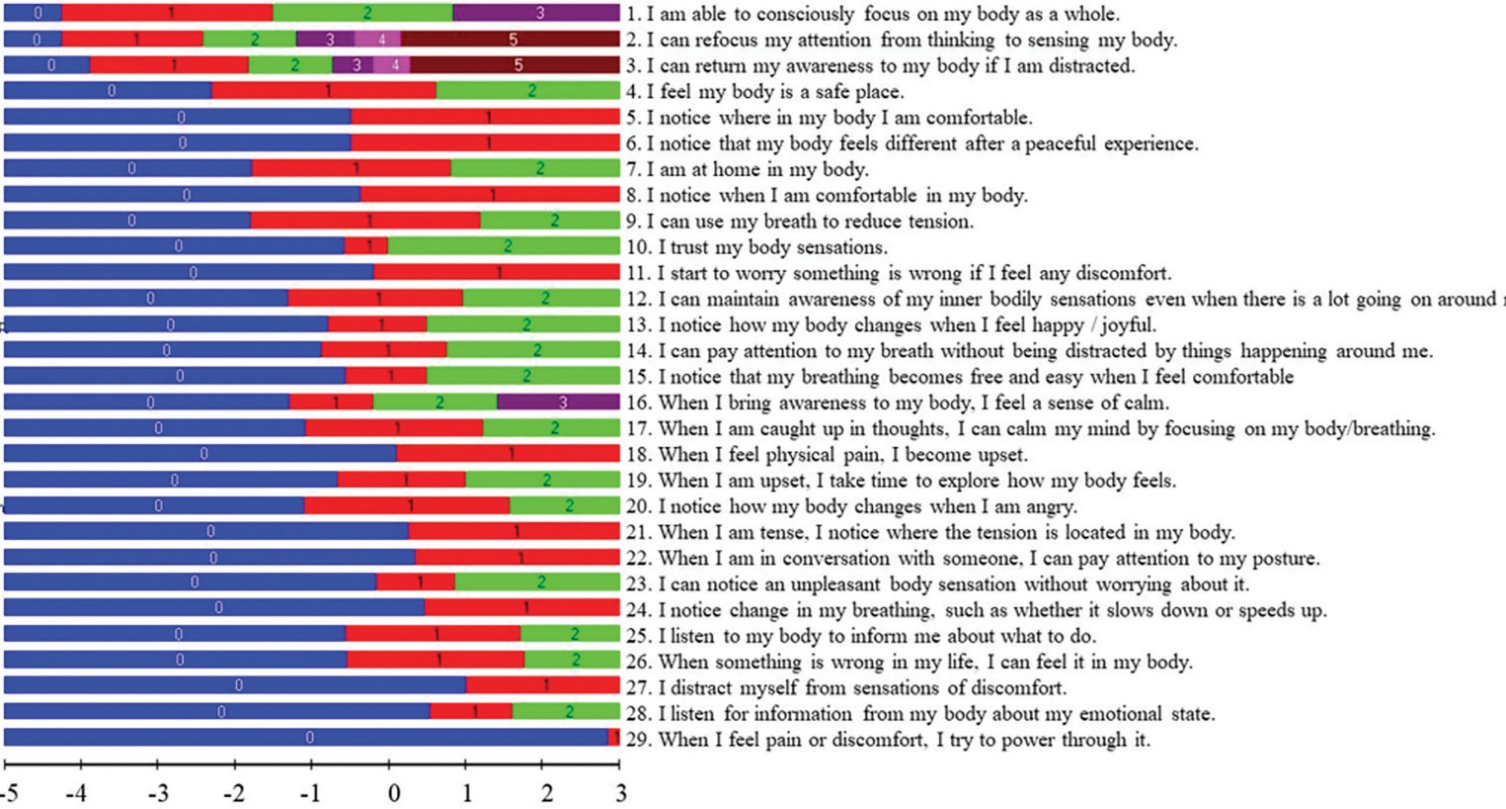
Item threshold map in adults with stroke. The easiest MAIA items are at the top and the hardest items are at the bottom of the list. The horizontal black line at the bottom is the logit ruler that can be used to show the location of the scoring options for each item as well as the person’s ability level of interoceptive body awareness (higher logit scores indicating higher body awareness ability).

The revised Rasch-based MAIA is shown in Table 2 and Fig 2 with items listed in order of difficulty on the logit scale from the easiest item at the top to the most difficult item at the bottom. In sum, the Rasch analysis resulted in a 29-item MAIA scale with a good overall fit, and good item and person fit.

**Table 2:** Rasch-based MAIA.

New Item Number	Never					Always
1. I am able to consciously focus on my body as a whole.	0	0	1	2	2	3
2. I can refocus my attention from thinking to sensing my body.	0	1	2	3	4	5
3. I can return my awareness to my body if I am distracted.	0	1	2	3	4	5
4. I feel my body is a safe place.	0	0	0	1	1	2
5. I notice where in my body I am comfortable.	0	0	0	0	1	1
6. I notice that my body feels different after a peaceful experience.	0	0	0	1	1	1
7. I am at home in my body.	0	0	0	1	1	2
8. I notice when I am comfortable in my body.	0	0	0	0	1	1
9. I can use my breath to reduce tension.	0	0	0	1	1	2
10. I trust my body sensations.	0	0	0	0	1	2
11. I start to worry something is wrong if I feel any discomfort.	0	0	0	0	1	1
12. I can maintain awareness of my inner bodily sensations even when there is a lot going on around me.	0	0	0	1	1	2
13. I notice how my body changes when I feel happy / joyful.	0	0	0	0	1	2
14. I can pay attention to my breath without being distracted by things happening around me.	0	0	0	1	1	2
15. I notice that my breathing becomes free and easy when I feel comfortable.	0	0	0	0	1	2
16. When I bring awareness to my body, I feel a sense of calm.	0	0	0	1	2	3
17. When I am caught up in thoughts, I can calm my mind by focusing on my body/breathing.	0	0	0	1	1	2
18. When I feel physical pain, I become upset.	0	0	0	0	1	1
19. When I am upset, I take time to explore how my body feels.	0	0	1	1	2	2
20. I notice how my body changes when I am angry.	0	0	0	1	1	2
21. When I am tense, I notice where the tension is located in my body.	0	0	0	0	1	1
22. When I am in conversation with someone, I can pay attention to my posture.	0	0	0	0	1	1
23. I can notice an unpleasant body sensation without worrying about it.	0	0	0	0	1	2
24. I notice change in my breathing, such as whether it slows down or speeds up.	0	0	0	0	1	1
25. I listen to my body to inform me about what to do.	0	0	0	1	1	2
26. When something is wrong in my life I can feel it in my body.	0	0	0	1	1	2
27. I distract myself from sensations of discomfort.	0	0	0	0	1	1
28. I listen for information from my body about my emotional state.	0	0	0	0	1	2
29. When I feel pain or discomfort, I try to power through it.	0	0	0	0	1	1

We report the score-to-measure in Table 3, which shows the conversion from ordinal total scores to logits to logit-based % from 0–100 for the total score. Our score-to-measure data provided should only be used with full data sets.

**Table 3:** Rasch-based MAIA score conversion.

Total MAIA Score	Logit	Converted to logits 0–100
0	-5.93	0.00
1	-5.00	8.66
2	-4.33	14.95
3	-3.84	19.51
4	-3.45	23.12
5	-3.13	26.09
6	-2.86	28.60
7	-2.63	30.76
8	-2.42	32.68
9	-2.24	34.39
10	-2.07	35.94
11	-1.92	37.37
12	-1.77	38.69
13	-1.64	39.91
14	-1.52	41.07
15	-1.40	42.16
16	-1.29	43.20
17	-1.18	44.20
18	-1.08	45.16
19	-0.98	46.08
20	-0.88	46.97
21	-0.79	47.83
22	-0.70	48.69
23	-0.61	49.52
24	-0.52	50.33
25	-0.43	51.13
26	-0.35	51.93
27	-0.26	52.72
28	-0.18	53.51
29	-0.09	54.30
30	-0.01	55.09
31	0.08	55.89
32	0.16	56.68
33	0.25	57.49
34	0.34	58.31
35	0.43	59.14
36	0.52	59.99
37	0.61	60.86
38	0.71	61.74
39	0.81	62.65
40	0.91	63.59
41	1.01	64.56
42	1.12	65.57
43	1.23	66.62
44	1.35	67.73
45	1.48	68.89
46	1.61	70.14
47	1.75	71.46
48	1.91	72.90
49	2.08	74.47
50	2.26	76.22
51	2.48	78.20
52	2.73	80.51
53	3.03	83.32
54	3.42	86.96
55	3.99	92.26
56	4.82	100.00

The overall fit of the model χ^2^(DF) = 74.59 (58), p = 0.07, as well as item fit, showed that the MAIA can assess interoceptive awareness as a unidimensional construct. The PCAR analysis showed an eigenvalue of 4.78 and a percentage variance of 16.49%, indicating that underneath the broader aspect of interoceptive awareness, the MAIA may encompass about 5 aspects of interoceptive awareness. The paired t-tests showed that the person location on the subtests created by the positive (items 2, 19, 20, 21, 22, 27, 28, 29) and negative (items 11, 12, 13, 14, 17, 30, 31) loadings on the first principal component showed that 21.95% of the person locations were significantly different between the subtests, confirming the underlying aspects of the MAIA. In the same vein, LID was found in 42 item pairs (S3 Table). The “Attention Regulation” and “Emotional Awareness” scales each contributed 14 pairs to LID.

## Discussion

The main results of this preliminary Rasch analysis in adults with stroke showed a 29-item MAIA scale with a good overall fit, and good item and person fit. Moreover, the MAIA was well-targeted for adults with chronic stroke. This result was obtained after extensive rescoring of the items, which may be explained for several different reasons.

First, when the MAIA items were developed, the original wording of question items asked respondents to rate “how true” an item was for them. However, after some pretests, this wording was found to be difficult and subsequently changed to “how often” this item was true for them [6]. Therefore, it makes sense that 9 of the 29 items we retained were maximally efficiently rescored in a dichotomous format, more like “True” or “False” questions. Additionally, using END form formatting (i.e., labeling extreme categories with unlabeled interim options) may have contributed to the necessity to merge several scoring categories [84, 85].

There is also debate in the research community as to whether combining positively and negatively worded items in the same scale may result in measurement inaccuracies [86, 87]. In our analysis, only 1 of the deleted reverse-scored items was negatively worded (item 5). However, several previous studies have omitted various negatively worded items from the “Not-Distracting” and “Not-Worrying” subscales for poor factor loading [42, 43, 46, 48, 52, 55].

Our results confirmed that the MAIA measures approximately 5 overarching aspects of interoceptive awareness as originally found by Mehling et al. (2012). The items that had a positive loading on the first principal component came primarily from the “Emotional Awareness” (EA) (items 19, 20, 21, 22) and “Body Listening” (BL) (items 27, 28, 29) subscales with one item from the “Noticing” subscale (item 2). This fits with the original finding from Mehling et al. (2012) of moderate correlations (r = .60) between EA and BL scales. Mehling et al. (2012) also specified that items from the EA and BL subscales were part of the same overarching dimension of “Mind Body Integration” (dimension 5) whereas item 2 (“I notice where I am tense in my body.”) is from the dimension of “Noticing” (dimension 1) also originally known as “Awareness of Body Sensations” [37].

The items with negative loadings on the first principal component contained primarily items from the “Attention Regulation” (AR) (items 11, 12, 13, 14, 17) and “Trusting” (TR) (items 30, 31) subscales. Mehling et al. (2012) originally found the AR and TR scales to also be moderately correlated (r = .50) [37]. The AR and TR subscales were originally found to measure 2 different areas of interoceptive awareness, the “Capacity to Regulate Attention” (dimension 3) and “Trusting Body Sensations” (dimension 4) aspects respectively [37]. In other words, our analysis of positive and negative loadings is consistent with 4 of the original dimensions purported to be measured by the MAIA excluding only the dimension of “Emotional Reactions and Attentional Response to a Sensation ’’ which contains all the originally reverse-scored items from the “Not-Worrying” and Not-Distracting” subscales. This overarching dimension may be measured by the MAIA, yet we cannot confirm this based on our analysis.

To the best of our knowledge, only one other previous study utilized the MAIA in adults with stroke, specifically to measure recovery of body awareness impairments after acute stroke recovery, and to identify the associations between body awareness impairments and sensation, motor impairment, self-efficacy, and quality of life after stroke [9]. The Body Perception Disturbance scale was positively associated with motor function/impairment, self-efficacy, and quality of life, meaning that greater body awareness was associated with better outcomes for these key outcomes [9]. In contrast, Serrada et al. (2021) found that body awareness as measured by the MAIA had a poor association with other measures utilized in their study including the NIHSS, the Functional Independence Measure, and the Body Perception Disturbance scale, and the Stroke Impact Scale [9, 70, 88–91]. Serrada et al. (2021) also mentioned that body awareness was reduced after stroke, with average scores being 14.10±6.34, 13.70±6.25, and 13.5±6.22, when MAIA was tested at 1, 3 and 6 months post-stroke respectively. Serrada et al. (2021) did not have their study participants fill out the MAIA at baseline (i.e., 1–14 days post-stroke) due to concerns of the MAIA being inappropriate to administer immediately after a stroke [9]. Serrada et al. (2021) did not recommend using the MAIA in adults after stroke due to the potential of the questions being too distressing for someone in the acute stroke phase and because their authors posited that neglect and hypo-vigilance would be more common after stroke than hypervigilance [9]. Serrada et al. (2021) proposed that the development of a measure of body awareness that is appropriate for use after acute stroke is necessary, and we agree. We did not encounter distress when acquiring the MAIA in our sample, but we recruited only adults with chronic stroke. Our scores were considerably higher (average of 25.17 ± 5.23) than the MAIA scores reported in Serrada et al. (2021) but our participants were between 6 months and 10.5 years post-stroke (with an average of 3.79±2.85 years post-stroke). Also, about 50% of our participants had past or current experience with breathing exercises and/or past body awareness training in their daily life and thus the type of questions asked on the MAIA might have been more familiar to some of them.

### Study limitations

Our preliminary Rasch results in our small sample are promising, but Rasch validation in a larger sample size is needed to confirm our findings. Also, we only recruited adults with chronic stroke and thus cannot generalize our findings to adults with acute or subacute stroke. A larger sample will also allow for DIF analysis and further psychometric analyses are needed such as evaluating sensitivity to change.

## Conclusion

Our preliminary Rasch-based MAIA indicates promising results for future use of the MAIA in adults with chronic stroke. Further studies are needed to validate the findings and complete the psychometrics on the MAIA.

## Supporting information

S1 Fig Category probability curve for Item 16 in adults with stroke. The category probability curve shows the probability of each category being selected on the Y-axis. The X-axis shows the item measured in logits demonstrating the person’s ability of their body awareness in relation to the question “I can maintain awareness of my whole body even when a part of me is in pain or discomfort”.
(TIF)

S2 Fig Category probability curve for item 23 in adults with stroke. The category probability curve shows the probability of each category being selected on the Y-axis. The X-axis shows the item measured in logits demonstrating the person’s ability of their body awareness in relation to the question: “When I feel overwhelmed, I can find a calm place inside”.
(TIF)

S1 Table Iteration table MAIA. (DOCX)

S2 Table Item fit statistics of the Rasch-based MAIA. (DOCX)

S3 Table Local item dependence (residual correlation ≥ 0.37). (DOCX)
