# Supplementary figures and images for: Preliminary Rasch analysis of the multidimensional assessment of interoceptive awareness in adults with stroke
Source: PLoS One. 2023 Jun 2;18(6):e0286657. doi: 10.1371/journal.pone.0286657 (PMC10237650; doi:10.1371/journal.pone.0286657)

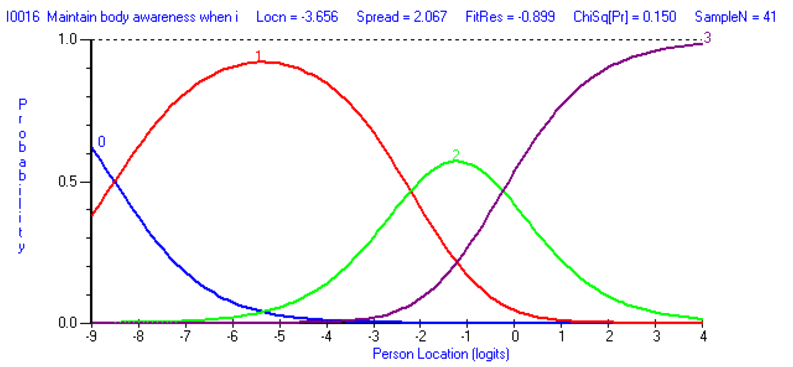

Supplement: S1 Fig — The category probability curve shows the probability of each category being selected on the Y-axis. The X-axis shows the item measured in logits demonstrating the person’s ability of their body awareness in relation to the question “I can maintain awareness of my whole body even when a part of me is in pain or discomfort”. (TIF) [file pone.0286657.s001.tif]

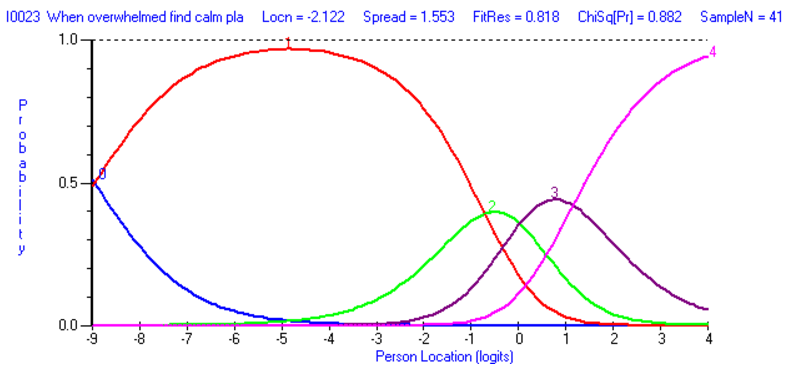

Supplement: S2 Fig — The category probability curve shows the probability of each category being selected on the Y-axis. The X-axis shows the item measured in logits demonstrating the person’s ability of their body awareness in relation to the question: “When I feel overwhelmed, I can find a calm place inside”. (TIF) [file pone.0286657.s002.tif]
