# Supplementary material for: Preliminary Rasch analysis of the multidimensional assessment of interoceptive awareness in adults with stroke
Source: PLoS One. 2023 Jun 2;18(6):e0286657. doi: 10.1371/journal.pone.0286657 (PMC10237650; doi:10.1371/journal.pone.0286657)
Supplement: S1 Table — (DOCX) [file pone.0286657.s003.docx]

**Table S1.** **Iteration table MAIA**

| **Analysis** | **Items** | **Rating scale groups** | **Person mean (SD) logits** | **Mean error variance** | **Floor effect n (%)** | **Ceiling effect n (%)** | **Overall Chi-square  (DF)  p-value** | **PSR** | **Number of items with disordered thresholds** | **Number misfitting Items (item #)** | **PCAR Eigenvalue 1st contrast (%)** | **Number of mis-fitting persons (%)** |
| --- | --- | --- | --- | --- | --- | --- | --- | --- | --- | --- | --- | --- |
| **All items**  (n = 41) | 32 | 192 | 0.55  (0.42) | 0.02 | 0 (0.00%) | 0  (0.00%) | 123.90  (64)  p = 0.00001 | 0.88 | 29 | 1 (7) | 5.68  (17.76%) | 5  (12.20%) |
| **Rescore** 27 items to [001234] | 32 | 165 | 0.33  (0.49) | 0.03 | 0 (0.00%) | 0  (0.00%) | 117.63  (64)  p = 0.00005 | 0.90 | 29 | 1 (7) | 5.67  (17.73%) | 3  (7.32%) |
| **Rescore** 22 items to [000123] | 32 | 143 | 0.25 (0.62) | 0.04 | 0 (0.00%) | 0  (0.00%) | 121.62  (64)  p = 0.00002 | 0.91 | 17 | 2 (5, 7) | 5.58  (17.44%) | 3  (7.32%) |
| **Rescore**  6 items to  [000011]  9 items to  [000012]  3 items to  [001223]  1 item to  [011223]  1 item to  [012233] | 32 | 114 | 0.21  (0.89) | 0.06 | 0 (0.00%) | 0  (0.00%) | 90.72  (64)  p = 0.02 | 0.92 | 6 | 0 | 5.10  (15.94%) | 2  (4.88%) |
| **Rescore**  3 items to  [000011]  2 items to  [000112]  1 item to  [001123] | 32 | 109 | 0.22 (0.94) | 0.07 | 0 (0.00%) | 0  (0.00%) | 94.19  (64)  p = 0.008 | 0.92 | 1 | 1 (5) | 4.86  (15.19%) | 2  (4.88%) |
| **Rescore**  1 item to [001122] | 32 | 108 | .25  (0.93) | 0.07 | 0 (0.00%) | 0  (0.00%) | 78.68 (62)  p = 0.008 | 0.93 | 0 | 1 (5) | 4.83  (15.11%) | 2  (4.88%) |
|  |  |  |  |  |  |  |  |  |  |  |  |  |
| **Delete**  Item 5 | 31 | 104 | 0.25  (1.05) | 0.08 | 0 (0.00%) | 0  (0.00%) | 78.68 (62)  p = 0.08 | 0.93 | 0 | 0 | 4.82  (15.55%) | 1  (2.44%) |
|  |  |  |  |  |  |  |  |  |  |  |  |  |
| **Delete**  Item 16 | 30 | 100 | 0.12  (1.03) | 0.083 | 0 (0.00%) | 0  (0.00%) | 74.38 (60)  p = 0.10 | 0.92 | 0 | 0 | 4.80  (16.01%) | 1  (2.44%) |
|  |  |  |  |  |  |  |  |  |  |  |  |  |
| **Delete** | 29 | 95 | 0.47 | 0.087 | 0 (0.00%) | 0  (0.00%) | 62.25 (58) | 0.92 | 0 | 0 | 4.73 | 1 |
| Item 23 |  |  | (1.03) |  |  |  | p = 0.33 |  |  |  | (16.31%) | (2.44%) |
| **Rescore**  5 items to [000112]  1 item to [000123] | 29 | 86 | 0.05 (1.12) | 0.11 | 0 (0.00%) | 0  (0.00%) | 70.16 (58)  p = 0.06 | 0.91 | 0 | 0 | 4.78  (16.48%) | 1  (2.44%) |
|  |  |  |  |  |  |  |  |  |  |  |  |  |
|  |  |  |  |  |  |  |  |  |  |  |  |  |
| **Rescore**  1 item to [000111]  2 items to [000112] | 29 | 85 | 0.05  (1.13) | 0.12 | 0 (0.00%) | 0  (0.00%) | 74.59 (58)  p = 0.07 | 0.91 | 0 | 0 | 4.78  (16.49%) | 1  (2.44%) |
|  |  |  |  |  |  |  |  |  |  |  |  |  |
|  |  |  |  |  |  |  |  |  |  |  |  |  |

Legend: RSG = Rating Scale Categories, PM = Person Mean Logits, SD = Standard Deviation, MEV = Mean Error Variance, FE = Floor Effect, CE = Ceiling Effect, DF = Degrees of Freedom, PSR = Person Separation Reliability, DT = Disordered Thresholds, PCAR = Principal Components Analysis of Residuals
