# Supplementary material for: Preliminary Rasch analysis of the multidimensional assessment of interoceptive awareness in adults with stroke
Source: PLoS One. 2023 Jun 2;18(6):e0286657. doi: 10.1371/journal.pone.0286657 (PMC10237650; doi:10.1371/journal.pone.0286657)
Supplement: S2 Table — (DOCX) [file pone.0286657.s004.docx]

**S2 Table. Item fit statistics of the Rasch-based MAIA**

| **Item number and Description of Rasch-based MAIA** | **Item location (logits)** | **SE** | **Fit Residuals** | **Chi-Square (DF=2)** | **p-value** |
| --- | --- | --- | --- | --- | --- |
| 1. I am able to consciously focus on my body as a whole. | -1.63 | 0.26 | 0.27 | 3.13 | 0.21 |
| 1. I can refocus my attention from thinking to sensing my body. | -1.62 | 0.18 | 0.22 | 1.18 | 0.55 |
| 1. I can return awareness to my body if I am distracted. | -1.26 | 0.17 | 1.62 | 0.25 | 0.88 |
| 1. I feel my body is a safe place. | -0.83 | 0.29 | -0.54 | 1.06 | 0.59 |
| 1. I notice where in my body I am comfortable. | -0.48 | 0.35 | -0.11 | 0.02 | 0.99 |
| 1. I notice that my body feels different after a peaceful experience. | -0.48 | 0.35 | -0.19 | 0.67 | 0.72 |
| 1. I am at home in my body. | -0.47 | 0.28 | 0.00 | 0.81 | 0.67 |
| 1. I notice when I am uncomfortable in my body. | -0.37 | 0.35 | -0.75 | 0.85 | 0.65 |
| 1. I can use my breath to reduce tension. | -0.29 | 0.29 | -0.46 | 0.51 | 0.78 |
| 1. I trust my body sensations. | -0.29 | 0.23 | -1.03 | 3.43 | 0.18 |
| 1. I start to worry that something is wrong if I feel any discomfort. | -0.19 | 0.35 | 2.45 | 7.79 | 0.02 |
| 1. I can maintain awareness of my inner bodily sensations even when there is a lot going on around me. | -0.16 | 0.27 | -0.40 | 3.17 | 0.21 |
| 1. I notice how my body changes when I am angry. | -0.13 | 0.24 | -0.75 | 1.24 | 0.54 |
| 1. I can pay attention to my breath without being distracted by things happening around me. | -0.05 | 0.25 | 0.50 | 1.03 | 0.60 |
| 1. I notice that my breathing becomes free and easy when I am comfortable. | -0.02 | 0.24 | 0.07 | 0.63 | 0.73 |
| 1. When I bring awareness to my body, I feel a sense of calm. | -0.01 | 0.21 | -1.67 | 6.18 | 0.05 |
| 1. When I am caught up in thoughts, I can calm my mind by focusing on my body/breathing. | 0.08 | 0.27 | -0.75 | 2.07 | 0.36 |
| 1. When I start to feel physical pain, I become upset. | 0.11 | 0.35 | 1.33 | 0.83 | 0.66 |
| 1. When I am upset, I take time to explore how my body feels. | 0.18 | 0.25 | 0.32 | 4.19 | 0.12 |
| 1. I notice how my body changes when I am angry. | 0.25 | 0.28 | -0.35 | 0.56 | 0.76 |
| 1. When I am tense, I notice where the tension is located in my body. | 0.26 | 0.35 | 0.12 | 0.12 | 0.94 |
| 1. When I am in conversation with someone, I can pay attention to my posture. | 0.35 | 0.35 | 0.77 | 2.17 | 0.34 |
| 1. I can notice an unpleasant body sensation without worrying about it. | 0.37 | 0.24 | 3.10 | 3.47 | 0.18 |
| 1. I notice changes in my breathing, such as whether it slows down or speeds up. | 0.48 | 0.35 | 0.12 | 1.09 | 0.58 |
| 1. I listen to my body to tell me what to do. | 0.59 | 0.27 | 0.02 | 0.26 | 0.88 |
| 1. When something is wrong in my life, I can feel it in my body. | 0.62 | 0.27 | 0.21 | 3.08 | 0.21 |
| 1. I distract myself from sensations of discomfort. | 1.02 | 0.37 | 0.84 | 11.68 | 0.00 |
| 1. I listen for information from my body about my emotional state. | 1.09 | 0.26 | 0.31 | 11.55 | 0.46 |
| 1. When I feel pain or discomfort, I try to power through it. | 2.86 | 0.59 | 0.61 | 11.61 | 0.11 |

Legend: SE = Standard Error, DF = Degrees of Freedom
