# Supplementary material for: Preliminary Rasch analysis of the multidimensional assessment of interoceptive awareness in adults with stroke
Source: PLoS One. 2023 Jun 2;18(6):e0286657. doi: 10.1371/journal.pone.0286657 (PMC10237650; doi:10.1371/journal.pone.0286657)
Supplement: S3 Table — (DOCX) [file pone.0286657.s005.docx]

**S3 Table. Local Item Dependence (Residual correlation ≥ 0.37)**

| Item number | RC | Scale |
| --- | --- | --- |
| 8. I notice when I am uncomfortable in my body. | 0.53 | NOT |
| 21. When I am tense, I notice where the tension is located in my body. |  | NOT |
| 5. I notice where in my body I am comfortable. | 0.42 | NOT |
| 21. When I am tense, I notice where the tension is located in my body. |  | NOT |
| 5. I notice where in my body I am comfortable. | 0.47 | NOT |
| 8. I notice when I am uncomfortable in my body. |  | NOT |
| 17. When I am caught up in my thoughts, I can calm my mind by focusing on   my body/breathing. |  | SR |
| 21. When I am tense, I notice where the tension is located in my body. |  | NOT |
| 11. I start to worry something is wrong if I feel any discomfort. | 0.49 | NW |
| 18. When I feel physical pain, I become upset. |  | NW |
| 12. I can maintain awareness of my inner bodily sensations even when there is a lot going on around me. | 0.59 | AR |
| 14. I can pay attention to my breath without being distracted by things happening around me. |  | AR |
| 12. I can maintain awareness of my inner bodily sensations even when there is a lot going on around me. | 0.58 | AR |
| 28. I listen for information from my body about my emotional state. |  | BL |
| 12. I can maintain awareness of my inner bodily sensations even when there is a lot going on around me. | 0.40 | AR |
| 25. I listen to my body to inform me about what to do. |  | BL |
| 3. I can return awareness to my body if I am distracted. | 0.55 | AR |
| 22. When I am in conversation with someone, I can pay attention to my   posture. |  | AR |
| 1. I am able to consciously focus on my body as a whole. | 0.39 | AR |
| 22. When I am in conversation with someone, I can pay attention to my   posture. |  | AR |
| 22. When I am in conversation with someone, I can pay attention to my posture. | 0.44 | AR |
| 26. When something is wrong in my life, I can feel it in my body. |  | EA |
| 22. When I am in conversation with someone, I can pay attention to my   posture. | 0.38 | AR |
| 25. I listen to my body to inform me about what to do. |  | BL |
| 1. I am able to consciously focus on my body as a whole. | 0.50 | AR |
| 3. I can return awareness to my body if I am distracted. |  | AR |
| 3. I can return awareness to my body if I am distracted. | 0.41 | AR |
| 20. I notice how my body changes when I am angry. |  | EA |
| 3. I can return awareness to my body if I am distracted. | 0.42 | AR |
| 26. When something is wrong in my life, I can feel it in my body. |  | EA |
| 3. I can return awareness to my body if I am distracted. | 0.44 | AR |
| 25. I listen to my body to inform me about what to do. |  | BL |
| 2. I can refocus my attention from thinking to sensing my body. | 0.38 | AR |
| 16. When I bring awareness to my body, I feel calm. |  | SR |
| 1. I am able to consciously focus on my body as a whole. | 0.37 | AR |
| 26. When something is wrong in my life, I can feel it in my body |  | EA |
| 1. I am able to consciously focus on my body as a whole. | 0.48 | AR |
| 28. I listen for information from my body about my emotional state. |  | BL |
| 20. I notice how my body changes when I am angry. | 0.37 | EA |
| 26. When something is wrong in my life, I can feel it in my body. |  | EA |
| 7. I am at home in my body. | 0.47 | TR |
| 20. I notice how my body changes when I am angry. |  | EA |
| 4. I feel my body is a safe place. | 0.42 | TR |
| 20. I notice how my body changes when I am angry. |  | EA |
| 13. I notice how my body changes when I am angry. | 0.51 | EA |
| 26. When something is wrong in my life, I can feel it in my body. |  | EA |
| 6. I notice that my body feels different after a peaceful experience. | 0.51 | EA |
| 13. I notice how my body changes when I am happy / joyful. |  | EA |
| 6. I notice that my body feels different after a peaceful experience. | 0.39 | EA |
| 28. I listen for information from my body about my emotional state. |  | BL |
| 6. I notice that my body feels different after a peaceful experience. | 0.54 | EA |
| 19. When I am upset, I take time to explore how my body feels. |  | BL |
| 6. I notice that my body feels different after a peaceful experience. | 0.37 | EA |
| 7. I am at home in my body. |  | TR |
| 4. I feel my body is a safe place. | 0.55 | TR |
| 6. I notice that my body feels different after a peaceful experience. |  | EA |
| 15. I notice that my breathing becomes free and easy when I feel comfortable. | 0.39 | EA |
| 19. When I am upset, I take time to explore how my body feels. |  | BL |
| 15. I notice that my breathing becomes free and easy when I feel comfortable. | 0.43 | EA |
| 25. I listen to my body to inform me about what to do. |  | BL |
| 7. I am at home in my body. | 0.46 | TR |
| 15. I notice that my breathing becomes free and easy when I feel comfortable. |  | EA |
| 7. I am at home in my body. | 0.39 | TR |
| 13. I notice how my body changes when I am angry. |  | EA |
| 4. I feel my body is a safe place. | 0.46 | TR |
| 13. I notice how my body changes when I am angry. |  | EA |
| 9. I can use my breath to reduce tension. | 0.42 | SR |
| 16. When I bring awareness to my body, I feel a sense of calm. |  | SR |
| 16. When I bring awareness to my body, I feel a sense of calm. | 0.47 | SR |
| 17. When I am caught up in my thoughts, I can calm my mind by focusing on my body/breathing. |  | SR |
| 9. I can use my breath to reduce tension. | 0.62 | SR |
| 17. When I am caught up in my thoughts, I can calm my mind by focusing on   my body/breathing. |  | SR |
| 19. When I am upset, I take time to explore how my body feels. | 0.37 | BL |
| 28. I listen for information from my body about my emotional state. |  | BL |
| 7. I am at home in my body. | 0.50 | TR |
| 19. When I am upset, I take time to explore how my body feels. |  | BL |
| 4. I feel my body is a safe place. | 0.48 | TR |
| 19. When I am upset, I take time to explore how my body feels. |  | BL |
| 4. I feel my body is a safe place. | 0.73 | TR |
| 7. I am at home in my body. |  | TR |
| 7. I am at home in my body. | 0.43 | TR |
| 10. I trust my body sensations. |  | TR |
| 4. I feel my body is a safe place. | 0.53 | TR |
| 10. I trust my body sensations. |  | TR |

Legend: RC (Residual Correlation), NOT (Noticing), ND (Not-Distracting), NW (Not-Worrying), AR (Attention Regulation), EA (Emotional Awareness), SR (Self-Regulation), BL (Body Listening), TR (Trusting)
